# Discovery of the Source of the Rochester Knockings

**Published:** 1851-03

**Authors:** 


### EDITORIAL DEPARTMENT.

### *Discovery of the Source of the Rochester Knockings.*

All our readers have heard of the *Rochester knockings* that have occasioned not a little stir in different parts of the country during the past two or three years. The *knockings* were first manifested in a family of the name of Fox, then residing in a small town in the western part of this State, and the removal of this family, shortly afterward, to Rochester, whence have emanated many of the marvelous stories connected with the subject, has secured for that city the honor of forming the adjective in the title by which they are commonly mentioned. The *knockings*, however, have not been confined to Rochester, but have been heard in some other places. They accompany members of the Fox family in their peregrinations, of course, but we understand that other persons than those belonging to this family have assumed to be *media* for similar supernatural manifestations.

Being regarded by the credulous and superstitious as phenomena produced by the agency of departed spirits, indicating their presence, and furnishing a means of communication with them, it is not singular that, however ridiculous the subject may seem to persons of well balanced minds, to those of a different mental cast, it assumes a different aspect, and becomes invested with great interest and importance. In every community persons are to be found who are fond of indulging and cultivating a love for what is marvelous, and who are ready to believe that a supernatural agency is involved in whatever transcends their comprehension. Such tendencies are by no means found in connection exclusively with low intellectual powers, and small attainments. On the contrary, it is not infrequently the case that persons of education, of reflection, and even of superior mental endowments in some respects, are led astray by what appeals strongly to the mental qualities underlying an unfortunate excess of credulity. The chicaneries of mesmerism, the faith inspired by revelations like those of Davis, etc., sufficiently attest the truth of the remark just made. We might also quote, as illustrations, the transient success of homœopathy, and other kindred medical delusions. The annals of every age furnish abundance of examples showing the absurd extravagances into which men may be led who allow unrestrained scope to the imaginative and superstitious elements of the mental constitution; showing, also, the astonishing extent to which cunning impostors are able to take advantage of these elements of human character. Based, as are the various delusions, impositions, and humbugs that prove successful, upon qualities of mind which it is not to be expected will soon cease to be predominant in certain individuals, albeit science and knowledge are progressively advancing, and despite the accumulated lessons of experience, we are not to suppose that the future, more than the past and present, will be devoid of instances exemplifying human weakness and folly like that to which reference has been made. But to return to the *Rochester knockings*. We have not taken pains to ascertain how extensively belief in their supernatural character has prevailed. Many of our readers are probably better informed on this point than ourselves, as our pursuits do not permit us to keep up with the times in matters of this kind. That many well meaning persons have been beguiled and carried away with this subject, we know, and that not a little time, money, thought, and feeling, have been expended in the efforts to hold communion, by *rappings*, with the inhabitants of the spirit world, is a fact but too apparent to any one who looks into newspapers. The imposition, unfortunately, is not to be considered merely a successful but harmless experiment on the exhaustless fund of human credulity. Among other serious consequences, we have been told that several cases of insanity have originated in the mental excitement occasioned by fancied intercourse with the spirits of departed friends.

The imposition, which had already escaped detection for several years, would still find plenty of dupes, if the *mysterious sounds* were to continue unaccounted for. The absurdity of the professed spirituality of the knockings can undoubtedly be fully proved in a variety of modes, but the only effectual preventive of the farther progress of the humbug is to determine satisfactorily their nature and source. To do this is to strike at the root of the delusion by rendering it as ridiculous as the explanation is simple. We are not aware that the curious and (in other than a literal sense) striking phenomena have been, as yet, accounted for. To what extent they have been made the subject of investigation by physicians we cannot say. As we are prepared to unravel the mystery, we trust our readers will not think the subject unworthy the space which we propose to devote to it, more especially as the sounds possess interest in a physiological point of view, apart from the remarkable imposition to which they have been made subservient.

Two members of the celebrated family of *Rochester knockers* recently made their debut in this city, accompanied by the noisy spirits, and commenced operations, drawing crowds of visitors at a dollar a head, many of whom were impressed with the wonderful revelations interpreted from the *raps*, and several intelligent persons became converts to the doctrine of the spiritual origin of the sounds. From motives of curiosity we were led, with our colleagues, to pay them a visit, and, we must confess, we were surprised and puzzled by the loudness of the sounds, the apparent evidences of non-instrumentality on the part of the females, and the different directions from which they seemed to emanate. Close observation, however, of the countenances and deportment of the two females, led to the conviction that the production of the sounds involved a voluntary effort by the younger sister of the two—a girl about seventeen years of age, the elder sister (who is said to be a widow) being about thirty-five. The latter was apparently the *managing partner*, conducting the spiritual communications, while the former, it was clear, was the performer, i. e. the one that produced the knockings. Assuming the above as a point of departure, by the process of reasoning given below, the diagnosis was, that the sounds must necessarily be articular. This conclusion and the process by which it was arrived at, were stated to a number of persons directly after the visit. The question, then, was how such sounds could come from joints. The snapping of the phalangal joints of one hand by lateral motions made with the other hand, is familiar to every one. Some persons have the power to produce the same snapping by means of the muscles inserted into the phalangal bones, without any aid from the other hand. Dislocated bones return to their place with an audible snap, as all surgeons know. A patient once consulted us for a loud noise in his joint produced by walking. Almost every one has occasionally, by an accidental oblique movement of the lower extremities, caused a loud report in the knee joint. These facts suggested themselves, but works on physiology, anatomy, and dislocations, were consulted, in vain, for any account of loud noises like the *Rochester knockings* originating in the articulations. While pursuing these inquiries, which had been unexpectedly provoked, we chanced to meet with a person who said that his wife could produce similar sounds. He did not then know in what way they were produced; his wife had, in jest, kept him in ignorance on this point. At our request he immediately went home to ascertain, and returned with the information that the noise came from the knee joint, and that we were at liberty to satisfy ourselves with respect to this fact, and also of the mode in which they were produced. Accordingly, at first alone, and afterward accompanied by Drs. Lee and Coventry, (in concert with whom the prior investigations were conducted,) we visited the lady referred to, and on the following day the subjoined exposition was communicated for one of the daily papers of the city.*

*To the Editor of the Commercial Advertiser:*

Curiosity having led us to visit the room at the Phelps House in which two females from Rochester, (Mrs. Fish and Miss Fox,) profess to exhibit *striking* manifestations of the spiritual world, by means of which communion may be held with deceased friends, &c., and having arrived at a physiological explanation of the phenomena, the correctness of which has been demonstrated in an instance that has since fallen under observation, we have felt that a public statement is called for, which may perhaps serve to prevent further waste of time, money, and credulity, (to say nothing of sentiment and philosophy,) in connection with this so long successful impositions

The explanation is reached almost by a logical necessity, on the application of a method of reasoning much resorted to in the diagnosis of diseases

---

* In transferring that communication to our columns, we have corrected an error in the account of the displacement of the joint which produces the sounds. The exposition was drawn up hastily, and published at once, in order to check as promptly as practicable the farther progress of the imposition, and the mechanism was not so fully ascertained, as it has been by subsequent examinations. We will thank editors of medical Journals who may notice this matter to copy the anatomical explanation from this article, and not from the newspaper, and to make the correction referred to, should they have already quoted the first statement.

viz: *reasoning by way of exclusion.* It was reached by this method prior to the demonstration which has subsequently occurred.

It is to be assumed, first, that the manifestations are not to be regarded as spiritual, provided they can be physically, or physiologically accounted for. Immaterial agencies are not to be invoked until material agencies fail. We are thus to *exclude* spiritual causation in this stage of the investigation.

Next, it is taken for granted that the *rappings* are not produced by artificial contrivances about the persons of the females, which may be concealed by the dress. This hypothesis is excluded, because it is understood that the females have been repeatedly and carefully examined by lady committees.

It is obvious that the rappings are not caused by machinery attached to tables, doors, etc., for they are heard in different rooms, and different parts of the same room, in which the females are present, but always *near* the spot where the females are stationed. This mechanical hypothesis is then to be *excluded.*

So much for *negative* evidence, and now for what *positively* relates to the subject.

On carefully observing the countenances of the two females, it was evident that the sounds were due to the agency of the younger sister, and that they involved an effort of the will. She evidently attempted to conceal any indications of voluntary effort, but in this she did not succeed:—a volutary effort was manifest, and it was plain that it [illegible]uld not be continued very long without fatigue.

Assuming, then, this positive fact, the inquiry arises, how can the will be exerted to produce sounds (rappings) without obvious movements of the body? The voluntary muscles are the only organs (save those which belong to the mind itself) over which volition can exert any direct control. But the contractions of the muscles do not, in the muscles themselves, occasion obvious sounds. The muscles, therefore, to develope audible vibrations, must act upon parts with which they are connected. Now, it was sufficiently clear that the rappings were not *vocal* sounds: these could not be produced without movements of the respiratory muscles, which would at once lead to detection. Hence, *excluding* vocal sounds, the only *possible* source of the noises in question, produced, as we have seen they must be, by voluntary muscular contractions, is in one or more of the movable articulations of the skeleton. From the anatomical connections of the voluntary muscles, this explanation remains as the only alternative.

By an analysis prosecuted in this manner, we arrive at the conviction that the *rappings*, assuming that they are not spiritual, are produced, by the action of the will, through voluntary muscles, upon the joints.

Various facts may be cited to show that the motion of joints, under certain circumstances, is adequate to produce the phenomena of the *rappings;* but we need not now refer to these. By a curious coincidence, after arriving at the above conclusion respecting the source of the sounds, an instance has fallen under our observation which demonstrates the fact that noises precisely identical with the *spiritual rappings* may be produced in the *knee joint.*

A highly respectable lady, of this city, possesses the ability to develope sounds similar both in character and degree to those professedly elicited by the Rochester imposters from the spiritual world. We have witnessed the production of the sounds by the lady referred to, and have been permitted to examine the mechanism by which they are produced. Without entering, at this time, into a minute anatomical and physiological explanation, it is sufficient to state that, owing to relaxation of the ligaments of the knee joint, by means of muscular action, and pressure of the lower extremity against a point of resistance, the large bone of the leg (the tibia) is moved laterally upon the lower surface of the thigh bone (the femur) giving rise, in fact, to partial lateral dislocation. This is effected by an act of the will, without any obvious movement of the limb, occasioning a loud noise, and the return of the bone to its place is attended by a second sound. Most of the Rochester rappings are also double. It is practicable, however, to produce a single sound, by moving the bone out of place with the requisite quickness and force and allowing it to slide slowly back, in which case it is noiseless.

The visible vibrations of articles in the room situated near the operator, occur if the limb, or any portion of the body, is in contact with them at the time the sounds are produced. The force of the semi-dislocation of the bone is sufficient to occasion distinct jarring of doors, tables, etc., if in contact. The intensity of the sound may be varied in proportion to the force of the muscular contractions, and this will render the apparent source of the rappings more or less distinct.

We have witnessed repetitions of experiments in the case just referred to, sufficient to exhibit to us all the phenomena of sounds belonging to the Rochester rappings, and without further explanations at this time, we append our names in testimony of the facts contained in the foregoing hastily penned exposition.

University of Buffalo. AUSTIN FLINT, M. D.,<br>CHARLES A. LEE, M. D.,<br>C. B. COVENTRY, M. D.

Feb. 17, 1851.

The disclosure announced in the foregoing communication occasioned not a little excitement among those who had become interested in the *knockings*. The correctness of the explanation was not only called in question by these, but was doubted by many who had not hesitated to look upon the matter as a gross deception. The Rochester *Ladies*, of course, stoutly denied the imputation that the sounds proceeded from the joints, or were produced by any agency of theirs, and, the next day, they inserted in the daily papers the following card:—

ROCHESTER KNOCKINGS.

*To Docts. Flint, Coventry and Lee:*

GENTS: We observe by a communication in the *Commercial Advertiser*, that you have recently made an examination of a highly respectable lady of this city, by which you have discovered the secret of the "Rochester Imposters." As we do not feel willing to rest under the imputation of being imposters, we are very willing to undergo a proper and decent examination, provided we can select three male and three female friends who shall be present on the occasion.

We can assure the public that there is no one more anxious than ourselves to discover the origin of these mysterious manifestations. If they can be explained on "anatomical" and "Physiological" principles, it is due to the world that the investigation be made, and that the "Humbug" be exposed. As there seems to be much interest manifested by the public on this subject, we would suggest that as early an investigation as is convenient would be acceptable to the undersigned.

ANN L. FISH.
MARGARETTA FOX.

The invitation thus proffered was accepted by those to whom it was addressed, and on the following evening, by appointment, the examination took place. After a short delay, the two Rochester females being seated on a sofa, the knockings commenced, and were continued for some time in loud tones and rapid succession. The "spirits" were then asked "whether they would manifest themselves during the sitting and respond to interrogatories." A series of *raps* followed, which were interpreted into a re. ply in the affirmative. The two females were then seated upon two chairs placed near together, their heels resting on cushions, their lower limbs extended, with the toes elevated and the feet separated from each other. The object in this experiment was to secure a position in which the ments of the knee joint should be made tense, and no opportunity offered to make pressure with the foot. We were pretty well satisfied that the displacement of the bones requisite for the sounds could not be effected unless a fulcrum were obtained by resting one foot upon the other, or on some resisting body.

The company, seated in a semi-circle, quietly waited for the "*manifestations*" for more than half an hour, but the "spirits," generally so noisy, were now dumb. The position of the younger sister was then changed to a sitting posture, with the lower limbs extended on the sofa, the elder sister sitting, in the customary way, at the other extremity of the sofa. The "spirits" did not choose to signify their presence under these circumstances, although repeatedly requested so to do. The latter experiment went to confirm the belief that the younger sister alone produces the *rappings.* These experiments were continued until the females themselves admitted that it was useless to continue them longer at that time, with any expectation of *manifestations* being made.

In resuming the usual position on the sofa, *knockings* very soon began to be heard. It was then suggested that some other experiment be made. This was assented to, notwithstanding the first was, in our minds, amply conclusive. The experiment selected was, that the knees of the two females should be firmly grasped with the hands so applied that any lateral movement of the bones would be perceptible to the touch. The pressure was made through the dress. It was not expected to prevent the sounds, but to ascertain if they proceeded from the knee joint. It is obvious that this experiment was necessarily far less demonstrative, to an observer, than the first, because if the bones were distinctly felt to move, the only evidence of this fact would be the testimony of those whose hands were in contact with them. The hands were kept in apposition for several minutes at a time, and the experiment repeated frequently, for the course of an hour, or more, with negative results: that is to say, there were plenty of *raps* when the knees were not held, and none when the hands were applied save once, as the pressure was intentionally somewhat relaxed, (Dr. Lee being the holder,) two or three faint, single *raps* were heard, and Dr. Lee immediately averred that the motion of the bone was plainly perceptible to him. The experiment of seizing the knees as quickly as possible when the knockings first commenced, was tried several times, but always with the effect of putting an immediate *quietus* upon the *manifestations.*

The proposition to bandage the knees was then discussed. This experiment was objected to, on the part of the friends of the females, unless we would concede that it should be an exclusive test experiment. We were not prepared with appliances to render the limb immovable, and therefore declined to have it considered such a test. This was the experiment anticipated, and one which, we presume, the females thought would end in their triumph. A bandage applied above and below the patella, admitting of flexion of the limb, will probably not prevent the displacement, as we have but little doubt had been ascertained by the Rochester females before an examination was invited. Should it become necessary to repeat experiments in other places, in furtherance of the explosion of the imposition, we would suggest that the bandage be not relied upon. Plenty of roller, with lateral splints, firmly applied, so as to keep the limbs extended, and render the joints immovable, would doubtless succeed in arresting sounds so far as they involve the knee joint. It will be observed that, in our exposition, we do not claim that this joint is exclusively the source of sounds, and had our experiments, which were first directed to this joint, failed, we should have proceeded to interrogate, experimentally, other articulations. This, however, as the reader will note, seemed quite unnecessary. The conclusion seemed clear that the *Rochester knockings* emanate from the knee joint.

Since the exposition was published, we have heard of several cases in which movements of the bones entering into other articulations are produced by muscular effort, giving rise to sounds. We have heard of a person who can develop knockings from the ankle, of several who can produce noises with the joints of the toes and fingers, of one who can render loudly audible the shoulder, and another the hip joint. We have also heard of two additional cases in which sounds are produced by the knee joint. We have not, as yet, had an opportunity to make a personal examination in any of these cases, or to hear the sounds. The exposure of the imposition opens a new and curious field of physiological inquiry, and we would commend the subject to those who have leisure and facilities for prosecuting it. *Articular* as well as *articulated* sounds seem to claim an investigation which they have not heretofore received. Had the facts which the detection of this trick has developed, been contained in anatomical or physiological treatises, the progress of the deception would have been arrested long ere this. Doubtless these facts are not entirely new—they must have been observed in other cases the histories of which have escaped record. That sounds so loud should originate in the way we have ascertained that they are produced, would surprise even the medical listener, and perhaps seem almost incredible. It is readily conceivable how to other than medical listeners, the phenomena should appear, not only inexplicable, but in a high degree mysterious. The remark was made by many after the explanation was pulished that it required almost as much stretch of the imagination to believe that such sounds could be produced in joints, as that they involved a supernatural agency. The anatomical conformation of the knee joint is evidently most favorable for the production of loudsoundsby displacement. The broad articular surfaces offer considerable space for lateral motion, provided the ligaments are sufficiently relaxed, and the requisite motor force is properly applied. The relative shortness of the outer condyle of the femur favors the outward displacement, and true dislocation in this direction would be likely to occur, were it not for the numerous strong ligaments which render this the strongest articulation in the body. Owing to the great protection afforded by the ligaments against injuries to which, from the position and relations of this joint, it is particularly exposed, dislocations are, in fact, very rare in their occurrence. The displacement occasioning the *knockings* is sufficient to remove the ridge of bone which divides the two articular surfaces of the upper extremity of the tibia, from its situation in the sulcus between the condyles of the femur, and to carry it, more or less, upon the surface of the outer condyle. This movement gives rise to the first sound, and the return of the bone to its place causes the second sound, which, in the *Rochester knockings*, generally follows quickly upon the first. We are unable to explain fully the precise mechanism by which the displacement is effected. In the case of the lady of this city who reproduces the *spiritual rappings*, the bone slips outward with very slight voluntary effort, and it is not easy, from her own account, or by manual exploration, to determine the particular muscles that are brought to bear upon the joint. In this case the displacement daily occurs, in bending the limb, when no effort is made to produce it, but, under these circumstances, it is not generally attended with much noise. The bone returns to its place directly the muscular effort which has produced the displacement ceases. To develop sound the displacement must take place with a certain quickness and force, and the latter may be graduated, in some measure, at will. A fulcrum of the foot appears also to be requisite as already stated. The lady just referred to is now able to produce the sounds in one knee only. In early life she had this power in both knees. From the number and volume of sounds produced, it is evident that both the knees of the Rochester rappers now in this city are endowed with sonorous powers. It might be supposed that the frequent repetitions of these displacements would produce after a time irritation and disease within the joint. In the case of the lady of this city they are followed by some soreness, but in early life, when she was in the habit of practising them daily more or less, she experienced no pain, nor any unpleasant consequences, and she was then able to develop louder sounds than she can at present. How rare are instances of that peculiarity in the condition of the joint which admits of the audible phenomena that have given origin to the new science of *spiritual rappings*, we are unable to say. That they are not common is evident from the fact that the Rochester imposture has eluded detection so long; and that instances of a similar idiosyncrasy do occur, is shown by the fact that several *rappers* have appeared in different parts of the country. It is a sad commentary on human nature that the latter should prefer to have adopted and carried on the imposition when they discovered their peculiar power, rather than disclose the secret, and thus put a stop to the progress of the deception. Mrs. P., the lady of this city, to whom we are much indebted for the means of establishing the exposure to the satisfaction of the public, thus, deserves honorable mention, and the thanks of the community. A difficulty with some persons who have visited the *Rochester rappers*, in believing the sounds to be articular arises from the idea that the *raps* come from different quarters of the room, at a distance from the place at which the females are stationed. This difficulty involves several explanatory circumstances. In the first place, the sounds do not really come from a distance. It may seem that this is so, but it is a delusion, arising from not appreciating correctly some of the laws of acoustics. We do not ordinarily determine the direction from which aural impressions are received, save by the conjoined exercise of other senses. Variations in the supposed distance of the source of sound may be imitated, simply by variations in intensity of the sound, provided the source be not obvious to other senses than hearing. Upon these principles the deceptions of the ventriloquist are based. The ventriloquist does not transmit his voice in different directions, and at various distances, as is vulgarly supposed, but he graduates its intensity so as to make it appear more or less remote, concealing, at the same time, all the external evidences that *he* makes the sounds, and he relies upon directing, by his conversation, the attention of the audience to particular places, for the success of his effort to make it appear that the sounds proceed from these places. The knee knockings are muffled by the dress, and the slight movements are also thus concealed; hence, females make the best impostors in this line. The *raps* are then conducted by whatever solid substances are in contact with the limb, or body. The *Rochester knockers* prefer that their visitors should be seated around a long table, they sitting at one extremity of the table. Placing the limb, then, in contact with any part of the table, the knockings seem to be upon the latter. But if the limb is in contact only with the floor, the sounds will appear to come from below. The Rochester females, when they wish to give exhibitions of the sounds, sometimes stand near a door. If they touch the door with a limb, or rest against it, the sounds seem to come from the door, and the door may be felt to vibrate. If they stand at a little distance from the door, the sounds appear to come from below. The *raps* do not, in reality, ever appear to come from much distance, unless the delusion is aided by a vivid imagination, or a degree of credulousness very easily operated on. The loudness of the sounds will, aside from the degree of motive power and quickness by which the displacement is effected, depend on the conducting properties of different bodies in contact.

That part of this scheme of imposition, which relates to the communications made by means of the *knockings*, opens a field of curious inquiry, not devoid of interest and importance. Admitting that the sounds are shown to be physically produced, and dependent on the volition of those engaged in conducting the deception, some, who have been impressed by the degree of penetration manifested in the accuracy of certain of the responses, and the striking character of tho fancied revelations, will ask, 'How are these phenomena to be accounted for ?' In accounts that have been published by many—we doubt not well-meaning and, on most subjects, sensible persons—there are statements which, to the reader who does not see fit to deny in *toto* the veracity and intelligence of the narrators, certainly must appear extraordinary. We do not propose to discuss at length this view of the subject. To do this does not belong to us, and would be inappropriate in the pages of a medical journal. We will offer but a few remarks.

Having traced the *knockings* to their source, explained the mechanism of their production, and thus divested them of their supernatural character and of all mystery, the field of inquiry just referred to presents an aspect different from that which it had prior to the exposition. While the origin of the sounds was unknown, the belief in their spiritual derivation would be entertained by those whose mental constitution and habits favored credulity in such matters, and the communications would be received with a corresponding degree of faith ; and even some, not over credulous persons, might reason themselves into the conviction that the sounds must be due to intelligent, invisible spirits, from the apparent utter impossibility of accounting, on any other hypothesis, for the information thereby obtained. But assuming that the deception is unmasked, and the mode in which it is conducted satisfactorily explained, it follows, of course, that the communications are part and parcel of the humbug, and it only remains to show how it is that they are of a character to occasion surprise and astonishment. This question might be disposed of, so far as the present subject is concerned, by saying that phenomena of the same character, and equally extraordinary, occur in connection with fortune-telling, into which it is not professed that spiritual agencies enter, and which no one supposes to involve aught beyond human sagacity. The question covers all the various modes of imparting pretended supernatural revelations.

Much is due to the laws of probabilities alone—in other words, many of the wonders are coincidencies, which always occur in a series of random guesses. This plain fact is not always recollected, viz : That whenever a response involves either an affirmative or negative, the chances that it will be right or wrong are exactly equal. Guesses under such circumstances, in the long run, will be as often true as false. It may be admitted, however, that the whole philosophy of the matter is not resolvable into the laws of probabilities: other reasons must therefore be given.—Several reasons suggest themselves, some of which we will mention, without attempting to assign to them, respectively, their precise force.

A person of close observation and great shrewdness can acquire a degree of skill in furnishing communications purporting to be spiritual, which can hardly be appreciated by one who has not given much thought to the subject. This is a kind of acquirement not sought for, except by those who mean to make it subservient to deception ; and, therefore, by most persons is but little understood. Let an individual of proper capacity, make it a business to study the significance of every slight movement, intonation of voice, and expression of countenance, as criteria of concealed thoughts, and let this pursuit be prosecuted for years, under the incentives aflorded by the love of gain or applause, and the fear of detection, and the tact thus acquired will be likely to develop results that appear almost incredible, and by the superstitious are regarded as divinations. This is one consideration to which not a little weight belongs.

Another explanatory consideration is as follows : Persons resorting to oracular communications, in proportion as their minds became excited, and full credence secured, can hardly fail to exhibit in various ways indications which are so many clues by which a practiced observer is led to apprehend facts supposed to be competely hidden. A person, who has been much interested in the *knockings*, and who believes that there exists a kind of mesmeric relation between the females and the questioners, by means of which the knowledge of the latter is perceived by the former, informed us that he observed those persons who had full faith that they should obtain true responses, generally got them, while those who were incredulous were unsuccessful. We do not doubt the correctness of this observation, and it is fully explained by reference to the consideration just stated.

They, too, who become converts, are anxious to explain any errors and incongruities in the Sibyline responses, and are ready to accept explanations which are only pertinent by a large latitude of construction. They have an eager desire that what they seek to have communicated shall be communicated, and are ready to adopt any kind of interpretation which will secure the credit of the spirit which condescends to hold intercourse with them. It is sufficiently obvious to those who have made the *art of discovering truth by observation* a subject of study, that a pre-conceived notion often gives a bias even to the exercise of the senses. Not a few of the *false facts* of science are thus derived. Persons are apt to see precisely what they have pre-determined they shall see. How much more is it to be expected that this self-deception will be operative, when, instead of the sober realities of scientific research, the credulous mind is in pursuit of information to be imparted by miraculous means !

Again, the impression produced by successful hits in any of the arts of soothsaying or conjuration, is naturally greater than is consistent with a due regard to the failures. The number of the latter is forgotten, while the former are remembered, and thus acquire an undue preponderance.—More especially this consideration will apply to the prodigies related in written narratives, taking cognizance of those things which are only wonderful when isolated. The principle is the same as that upon which certificates of secret nostrums appeal to the confidence of the public. Admitting the certificates to be authentic, and even true in point of fact, we have only the extraordinary cures, without any of the host of cases in which the effect of the remedy was either nugatory or pernicious. These cases may predominate immensely over those in which benefit was attributed, while the latter, if considered exclusively, seem to furnish an overwhelming mass of evidence. We might add to these considerations, others ; but we have already said more upon this branch of the subject than we had intended, and perhaps more than the indulgence of our readers will lead them to excuse. We must offer as an apology for according to the subject so much space, in addition to the reasons before assigned, the personal interest in it growing out of the part we have taken in the detection and disclosure of the source of the Rochester Knockings. In engaging in this investigation, we literally followed the scriptural injunction, to "believe not every spirit, but try the spirits." The result is an exposition, the correctness of which rests, in the first place, upon a train of reasoning which we claim to be in itself conclusive ; and, in the second place, upon demonstrative evidence, tested by experiments which may be readily repeated and extended in all places where the *knockings* may be re-produced. It remains to see whether this result will succeed in bringing the career of this singular species of imposture to a close, and thu s to say the least, diverting the current of credulity into some new channel.

Certain physical phenomena in addition to the knockings, are said to be occasionally produced in connection with the latter; such as moving of tables and chairs; opening and shutting bureau drawers; pulling the hair, etc., of persons assembled to witness the exhibition, and various other palpable demonstrations of what is claimed to be an unknown and mysterious agency. With regard to these phenomena we have only to say that none of them have fallen under our observation, nor are we aware that any have, as yet, been exhibited in this city, although we understand it has been intimated that they will appear by and by. Assuming that such phenomena do take place, we leave for others the task of explaining the mechanism by which they are produced.

